# Juvenile leg autotomy predicts adult male morph in a New Zealand harvestman with weapon polymorphism

**DOI:** 10.1093/beheco/arad029

**Published:** 2023-05-11

**Authors:** Erin C Powell, Christina J Painting, Glauco Machado, Gregory I Holwell

**Affiliations:** Te Kura Mātauranga Koiora/School of Biological Sciences, University of Auckland, 3a Symonds St, Auckland 1010, New Zealand; Te Aka Mātuatua School of Science, University of Waikato, Gate 8, Hillcrest Road, Hamilton 3240, New Zealand; LAGE do Departamento de Ecologia, Instituto de Biociências, Universidade de São Paulo, Rua do Matão, Travessa 14, no. 101, Cidade Universitária, São Paulo CEP 05.508-090, Brazil; Te Kura Mātauranga Koiora/School of Biological Sciences, University of Auckland, 3a Symonds St, Auckland 1010, New Zealand

**Keywords:** alternative reproductive tactic, carryover effect, conditional threshold, Opiliones, predation

## Abstract

Intraspecific weapon polymorphisms that arise via conditional thresholds may be affected by juvenile experience such as predator encounters, yet this idea has rarely been tested. The New Zealand harvestman *Forsteropsalis pureora* has three male morphs: majors (alphas and betas) are large-bodied with large chelicerae used in male–male contests, while minors (gammas) are small-bodied with small chelicerae and scramble to find mates. Individuals use leg autotomy to escape predators and there is no regeneration of the missing leg. Here, we tested whether juvenile experience affects adult morph using leg autotomy scars as a proxy of predator encounters. Juvenile males that lost at least one leg (with either locomotory or sensory function) had a 45 times higher probability of becoming a minor morph at adulthood than intact juvenile males. Leg loss during development may affect foraging, locomotion, and/or physiology, potentially linking a juvenile’s predator encounters to their final adult morph and future reproductive tactic.

## Teaser

Males of New Zealand harvestmen display drastic variation in body size and weapon form. Instead of fighting, small males must find unguarded female to mate with. These harvestmen can drop their legs to escape predators but once dropped, the legs never grow back. Male harvestmen that drop their legs during development were more likely to end up as small males at adulthood. Predator encounters as juveniles may shape a male’s mating strategy for life.

## INTRODUCTION

Competition for mates is the most important selective pressure favoring the evolution of exaggerated male weapons, such as horns and antlers. These weapons function during male–male contests to force the rival to withdraw (Emlen 2008; McCullough et al. 2016; Rico‐Guevara and Hurme 2019). Recently, complex intraspecific male polymorphisms in weapon size and shape have been described for several terrestrial arthropods, including insects (Rowland and Emlen 2009; Kelly and Adams 2010; Iguchi 2013; Matsumoto and Knell 2017) and arachnids (Buzatto and Machado 2014; Painting et al. 2015; Powell et al. 2020). Both genetic polymorphism and conditional thresholds, usually corresponding to nutritional resources acquired during development, are known to underlie male weapon polymorphism (Emlen and Nijhout 1999; Tomkins and Moczek 2009; Gotoh et al. 2011; Rowland et al. 2017).

Males of large-bodied morphs possessing large weapons may allocate resources differentially among traits to maximize their fitness. Trade-offs between exaggerated weaponry and other traits have been demonstrated in several taxa. These trade-offs include reduced investment in testes size (Simmons and Emlen 2006; Simmons et al. 2007; Somjee et al. 2018b), reduced somatic traits (e.g., eyes or wings) developing in proximity to weapons (Nijhout and Emlen 1998), compromised locomotory performance (Basolo and Alcaraz 2003; Goyens et al. 2015), and greater metabolic costs to maintain muscle associated with large weaponry (Bywater et al. 2014; Somjee et al. 2018a; O’Brien et al. 2019). However, such trade-offs are not consistent across all taxa. For instance, in some species, testes size is not negatively correlated with weapon size (Malo et al. 2005; Munguía-Steyer et al. 2012), larger males pay few locomotory costs (McCullough and Emlen 2013; McCullough and Tobalske 2013), males bear disproportionately larger weaponry at a lower relative metabolic cost (Somjee et al. 2021), or males develop larger compensatory traits, such as wings, that support locomotion despite their large weaponry (Painting and Holwell 2013). In contrast, males of the small-bodied morph do not invest in weaponry and may maximize their fitness by adopting alternative reproductive tactics (ARTs); thus, their form of mating acquisition is different from their larger conspecific counterparts (reviewed in Gross 1996; Oliveira et al. 2008). Despite some knowledge of the proximate mechanisms behind the development of male polymorphisms, there is still much to understand about how multiple male morphs evolve within populations.

While most studies on carry-over effects (i.e., how juvenile experience influences adult phenotype) focus on juvenile nutrition (e.g., Emlen and Nijhout 1999; Tomkins and Moczek 2009; Gotoh et al. 2011), other juvenile experiences, such as non-lethal injuries caused by interactions with predators, may be important. To escape from a predator attack, individuals of several animal groups employ limb autotomy, defined as the voluntary shedding of a body part at a pre-defined breakage plane when a stimulus is applied (Emberts et al. 2019). Numerous species across diverse taxa have evolved the ability to drop a leg, claw, or tail, conferring the immediate advantage of escaping an attack (Maginnis 2006a; Fleming et al. 2007). Limb autotomy occurs in some animals exhibiting male polymorphism and alternative reproductive tactics, such as lizards that lose their tail (e.g., Calsbeek and Sinervo 2008), crabs that lose their claws (e.g., Shuster 2008), and long-legged harvestmen (Arachnida: Opiliones) that lose their legs (e.g., Powell et al. 2021a). In comparison to lizards and crabs, harvestmen are unique in that they are unable to regrow the lost limb (Gnaspini and Hara 2007). This means that resources and energy are not expended in regeneration, but also that other potential costs of autotomy are retained across the lifetime. The costs of leg loss in harvestmen include reduced locomotor performance (Guffey 1999; Houghton et al. 2011; Escalante et al. 2013; Domínguez et al. 2016), reduced foraging ability (Guffey 1999), and a disadvantage in male–male contests for territory possession (Macías-Ordóñez 1997).

**Figure 1:**
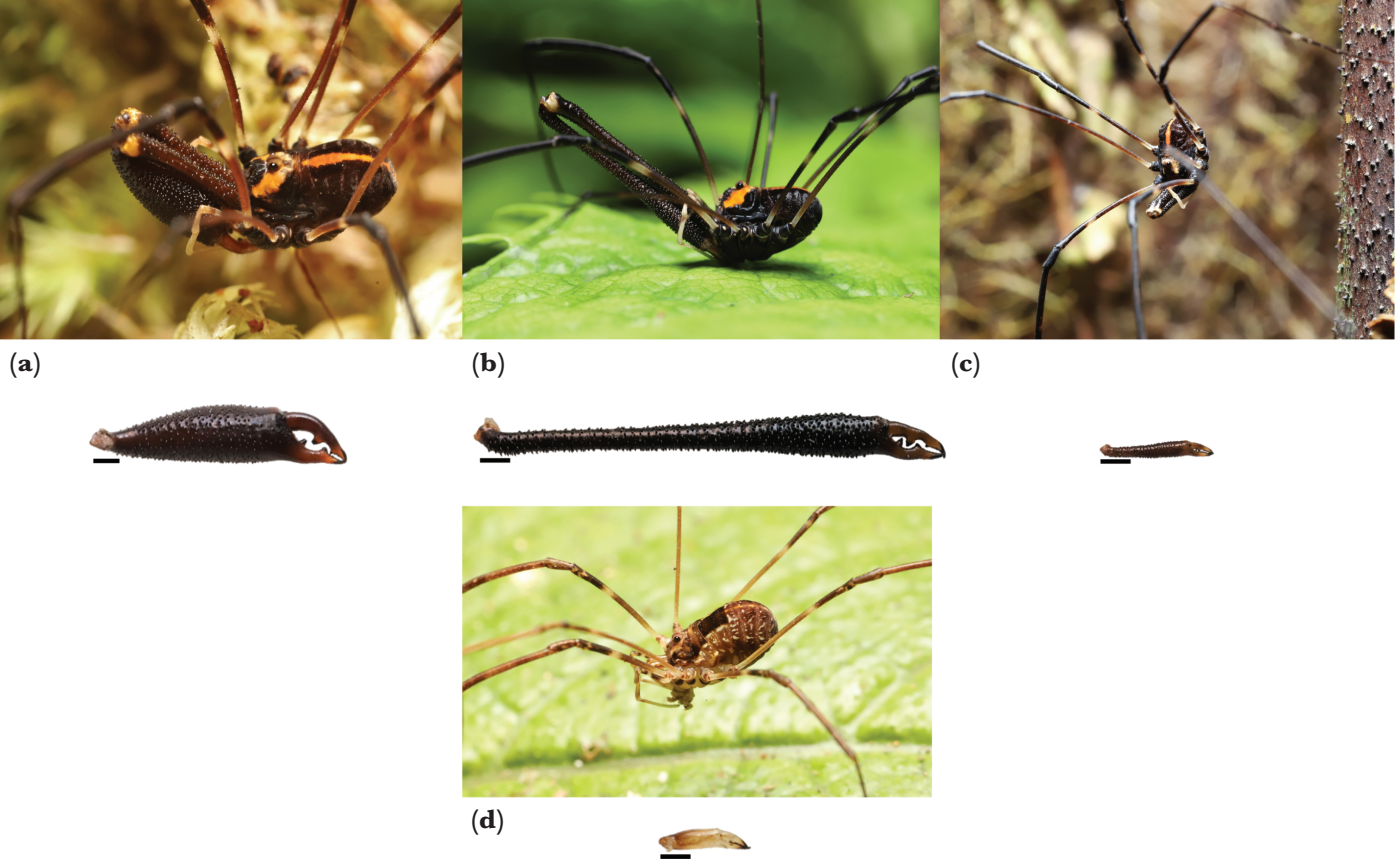
Intraspecific variation in the New Zealand harvestman *Forsteropsalis pureora*: (a) alpha male (major), (b) beta male (major) (c) gamma male (minor), and (d) female. The second cheliceral segment representative of alphas, betas, gammas, and females is shown underneath the corresponding *in situ* photograph. Scale bars indicate 1 mm. Photographs by E.C. Powell.

**Figure 2:**
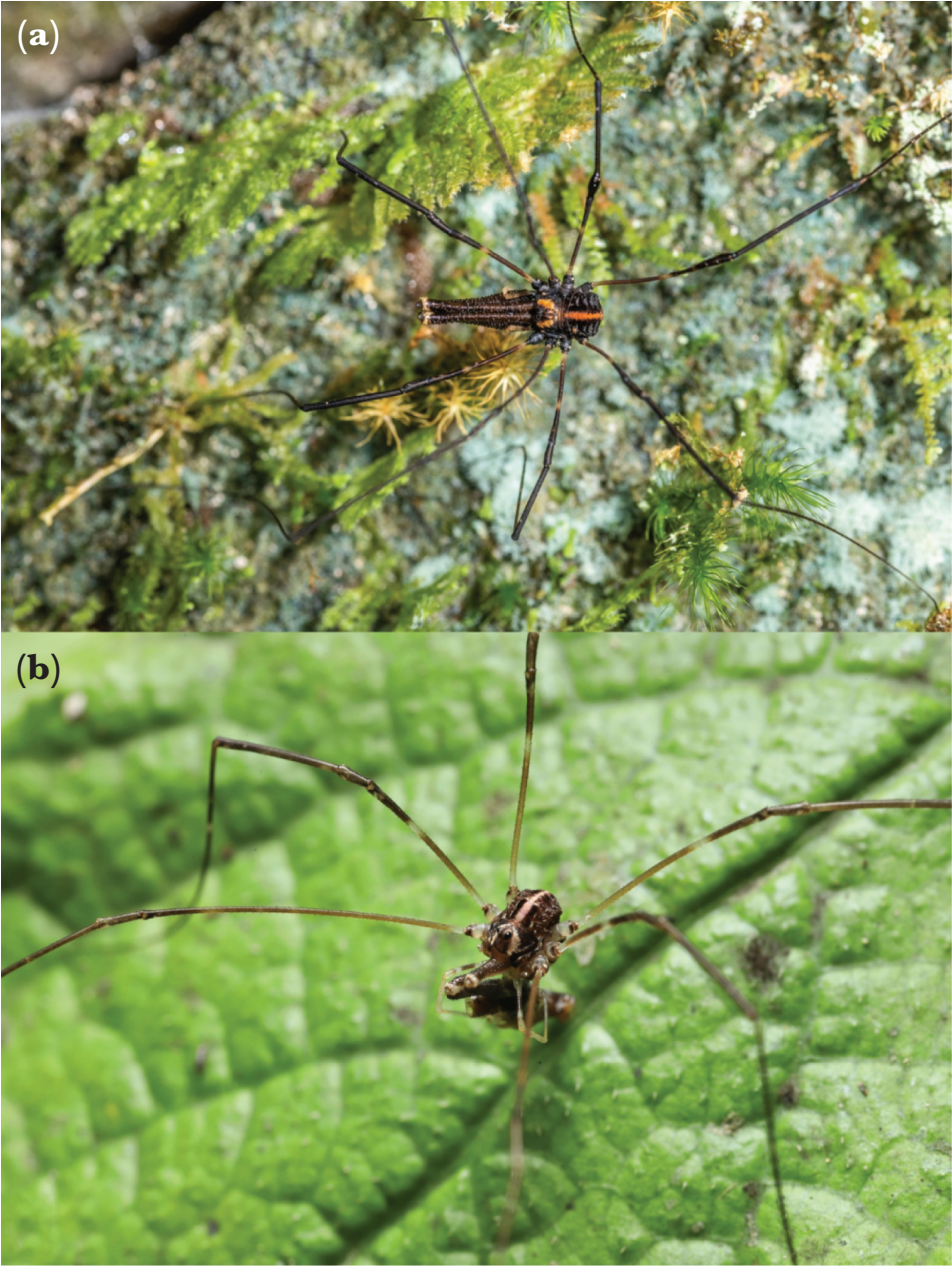
(a) A dorsal view of a major morph male *Forsteropsalis pureora* with fully intact legs. Note that the Legs II are exclusively sensory, not used for locomotion, and longer than the rest of the legs. Photograph by D. Hegg. (b) A minor morph male *F*. *pureora*, with both Legs I autotomized as a juvenile. This male is foraging on scavenged prey. Photograph by E. C. Powell.

The New Zealand harvestman *Forsteropsalis pureora* Taylor, 2013 (Neopilionidae) exhibits male trimorphism, with male morphs differing in body size, and in the size and shape of their weaponry (Powell et al. 2020). The morphs include a small-bodied male with tiny chelicerae (gamma), a large-bodied male with long-slender chelicerae (beta), and a large-bodied male with short but broad chelicerae (alpha), all of them coexisting within single populations (Figures 1 and 2). Alpha and beta males can have a body mass up to seven times higher than that of gamma males, demonstrating the drastic intraspecific variation found in this species. Gamma males adopt a scrambling strategy, searching through their environment to find mates and avoiding contests with other males, while alpha and beta males use their exaggerated chelicerae as weapons in contests to access females (Painting et al. 2015; Powell et al. 2020). We previously found that leg autotomy is a common defence strategy in many neopilionid species from New Zealand (Powell et al. 2021a), but adult male morphs do not experience different rates of autotomy despite their different reproductive tactics (Powell et al. 2021b).

Because harvestmen autotomize legs across juvenile and adult stages, with no regeneration, leg loss may influence which morph a male can develop into at adulthood. If legs are lost at the juvenile stage, males may have reduced abilities to search for, detect, and capture food compared to conspecifics with intact legs, which may prevent them from obtaining the necessary resources required to reach the conditional threshold for a large-bodied male morph (Tomkins and Hazel 2007). Moreover, a male that has lost legs as a juvenile may benefit from investing more in post-copulatory traits (e.g., testes size to increase fertilization success in leaf-footed bugs, see Joseph et al. 2018 and Cirino et al. 2021). Finally, if intact legs are important for success during male-male contests for territory possession (e.g., harvestmen: Macías-Ordóñez 1997; leaf-footed bugs: Emberts et al. 2018), autotomized juvenile males may have higher chances of copulation as adults by adopting ARTs that do not involve monopolization of mates, thereby doing “the best of a bad job” (Dawkins 1980).

The type of leg lost during development may also influence final adult morph because different legs play different roles in harvestmen (Guffey 1998, 1999; Sensenig and Shultz 2006; Willemart et al. 2009). The second pair of legs (legs II) is entirely sensory and also known as antenniform legs (Willemart et al. 2009). The first pair of legs (legs I) are used as locomotor appendages, but also have sensory functions (Willemart et al. 2009). Finally, the third and fourth pair of legs (legs III and IV) are used exclusively as locomotor appendages; in some species legs IV bear large weaponry, such as spines and tubercles, but that is not the case for any neopilionid harvestman, including our study species *F*. *pureora* (Buzatto and Machado 2014). Whereas the loss of a sensory leg may hamper food detection, the loss of locomotory legs may hamper food search. Although the relative impact of losing a sensory or a locomotory leg cannot be easily anticipated, previous studies indicate that the negative effects of leg autotomy on foraging success depends on the type of missing leg (e.g., Escalante et al. 2013).

Here, we tested the hypothesis that leg loss during development would impact adult male morph in the trimorphic harvestman *F*. *pureora*. We predicted that juveniles that molt to adulthood with missing legs have a higher chance of developing into gamma males, while juveniles that molt to adulthood with fully intact legs have a higher chance of developing into alpha or beta males. We also explored whether the type of leg lost during development may influence male morph at adulthood. Our dataset includes adult male harvestmen collected directly from the field. For each male missing at least one leg we recorded each autotomy as occurring as an adult or as a juvenile, as well as the type of leg lost.

## METHODS

Adult males (*N* = 86) of *F*. *pureora* were collected between Waitomo and Te Anga, New Zealand, across four sites: Ruakuri Bushwalk, Waitomo (38°15ʹ53.7ʹʹS 175°04ʹ46.4ʹʹE); private land, Te Anga (38°15ʹ41.4ʹʹS 175°00ʹ53.6ʹʹE); Tawarau Forest, Te Anga (38°17ʹ24.8”S 174°56ʹ50.2ʹʹE); and Marokopa Falls Track, Te Anga (38°15ʹ33.6ʹʹS 174°50ʹ54.6ʹʹE). Individuals were hand collected between 21:00 and 02:00 hours in January and February 2018. They were then contained in individual large vials and transported to the field laboratory, where they were euthanized and stored in 95% ethanol.

Sexual maturity was confirmed by the presence of a functional genital operculum, which is fused during the juvenile stage, but can be opened upon the adult molt (Gnaspini 2007). Following Powell et al. (2020), we assigned each male to either major morph (alphas and betas, *N* = 63) or minor morph (gamma, *N* = 23). Morph frequency in our sample, with about 75% of majors and 25% of minors, closely followed that of the larger sample in Powell et al. (2020). Males were randomly collected but it is possible that fewer minors were located because they were moving cryptically on the forest floor rather than resting on top of vegetation, as occurs with majors. We did not consider alphas and betas separately because, according to our hypothesis, both morphs probably had access to a greater amount of food resources during development. Moreover, Painting et al. (2015) hypothesized that trimorphism in New Zealand neopilionids arises via both a genetic polymorphism (which promotes the existence of two major morphs) and polyphenism (which promotes the existence of majors and minors via environmental conditions during development). Therefore, because leg loss during development should act exclusively on the polyphenic mechanism, our data consider specifically the dichotomy between majors and minors.

**Figure 3:**
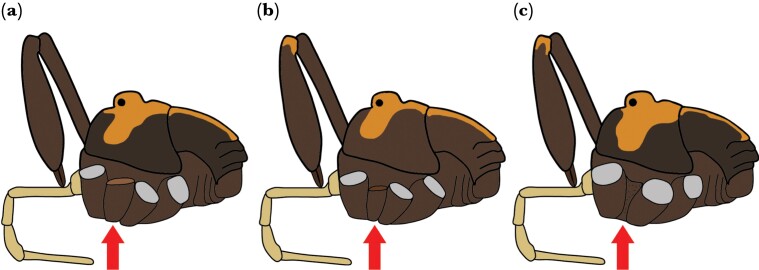
An illustrated example of the types of scars (see arrows) when adult and juvenile autotomy occurs in the legs of male *Forsteropsalis pureora*. (a) An adult male with new autotomy that occurred during adulthood. (b) An adult male with an autotomy that occurred as a juvenile. (c) An adult male with an autotomy that occurred even earlier as a juvenile, given how flat the scar is and that there are associated setae.

**Figure 4:**
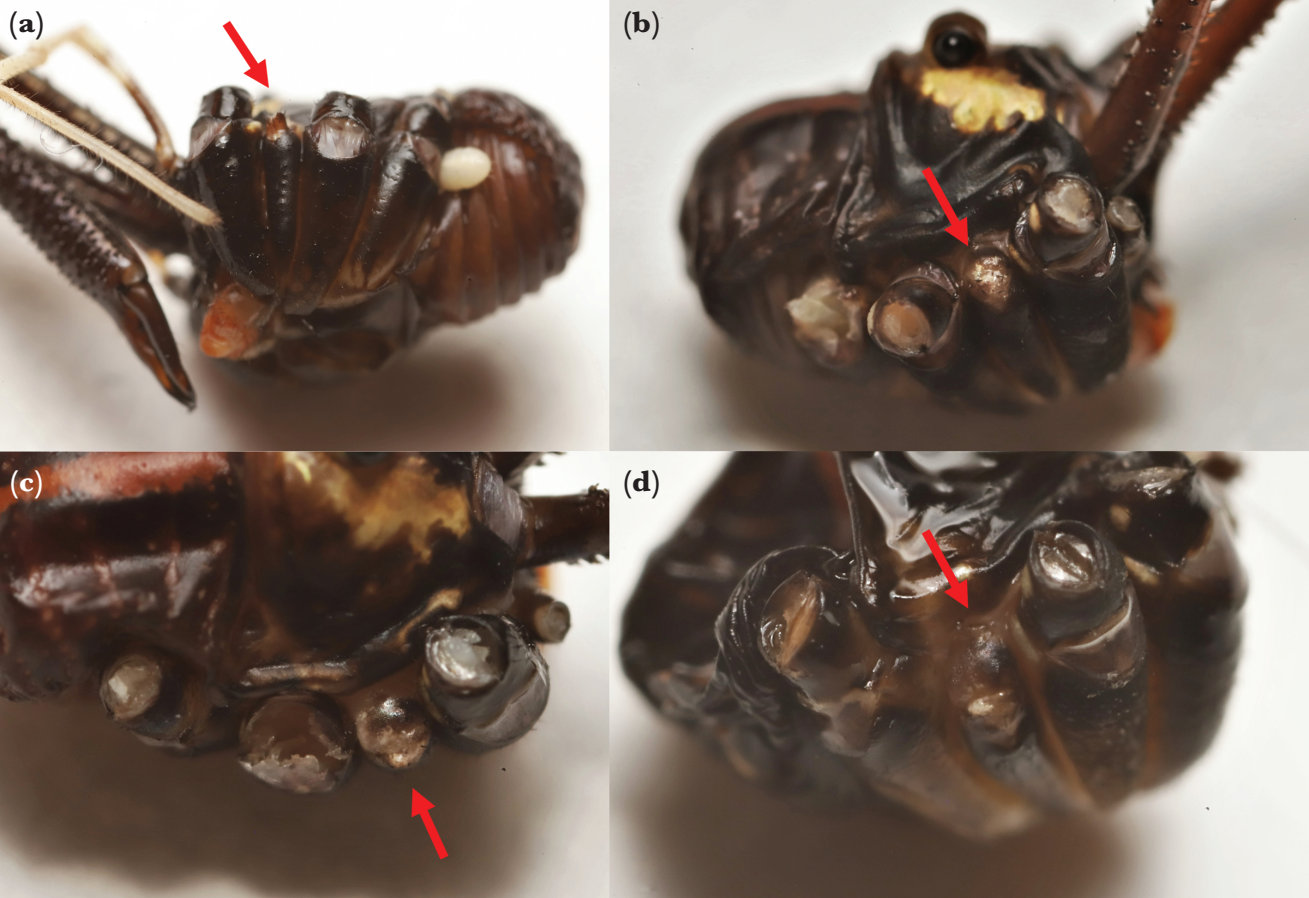
Examples of the types of scars (see arrows) remaining when adult and juvenile autotomy occurs in the legs of four males of the New Zealand harvestman *Forsteropsalis pureora*. For comparative purposes, we selected males that autotomized a leg of the second pair. (a) Major morph with a new autotomy that occurred during adulthood. Note that the trochanter of the autotomized leg has approximately the same size and shape as the non-autotomized legs. (b–d) Minor morphs with old autotomies that occurred as juveniles. Note the highly compressed coxa in (b) and associated setae on the scars in (c). Figure (d) may indicate autotomy that occurred in an even earlier instar because of how flat the scar is, but we were unable to determine the exact time during juvenile development that autotomy occurred.

To determine the “age” of autotomy, we inspected the remaining trochanter and coxa of the specimens that had experienced autotomy. Because harvestmen do not regenerate legs, scars remain at the location of legs lost across the individual’s life, even if the leg was autotomized early on in development. Thus, it is possible to tell if legs were autotomized prior to or after the last molt to adulthood (R. Macías-Ordóñez, personal communication). Fresh scars representing autotomy *during adulthood* were characterized by a fully intact trochanter (Figure 3a). If autotomy occurred *before adulthood*, the trochanter was reduced or even absent, with a sclerotized patch of cuticle over the location where the trochanter joint used to be (Figures 3a,b and 4a–d). Further, this patch of cuticle sometimes had associated setae (Figures 3c and 4b,c) and in some specimens the coxa joint of the missing leg was highly reduced and compressed by the coxa of the intact legs (Figures 3c and 4a), making it clear that legs were autotomized at least one molt before adulthood (E.C. Powell, personal observation). For the 50 individuals that experienced *any* autotomy, we classified scars as *recent*, if they occurred during adulthood, or *old*, if they occurred during development, i.e., as a juvenile at least one molt before adulthood.

To test whether leg loss (yes or no) during development correlates with adult male morph, we generated a generalized linear model (GLM) with a binomial response variable (logit link function) describing male morph identity: small-bodied minor morph (gamma) and large-bodied major morphs (alpha and beta). We did not include the number of missing legs in the model because only two males in our sample had more than one juvenile autotomy (see “Results”). We also tested whether the type of leg autotomized during development affects adult male morph (response variable) using a GLM with binomial distribution (logit link function). We used leg type as a predictor because, as mentioned above, legs serve different functions in harvestmen. The predictor variable “leg type” was divided into two levels: (a) locomotory legs, which includes legs III and IV, and (b) sensory legs, which includes legs II (Willemart et al. 2009). Legs I are used both as a locomotory and a sensory appendage (Willemart et al. 2009). Thus, to test the sensitivity of our results to the classification of legs I, we ran two separate GLMs, first classifying them as a locomotory and then as a sensory leg. As mentioned above, only two males had more than one juvenile autotomized leg in our sample, and thus it was not possible to include male identity as a random factor in the analyses. To avoid recording the multiple autotomies of these two males as independent points, we removed them from the analyses. All statistical analyses were performed in the software R (R Core Team 2020).

## RESULTS

**Figure 5:**
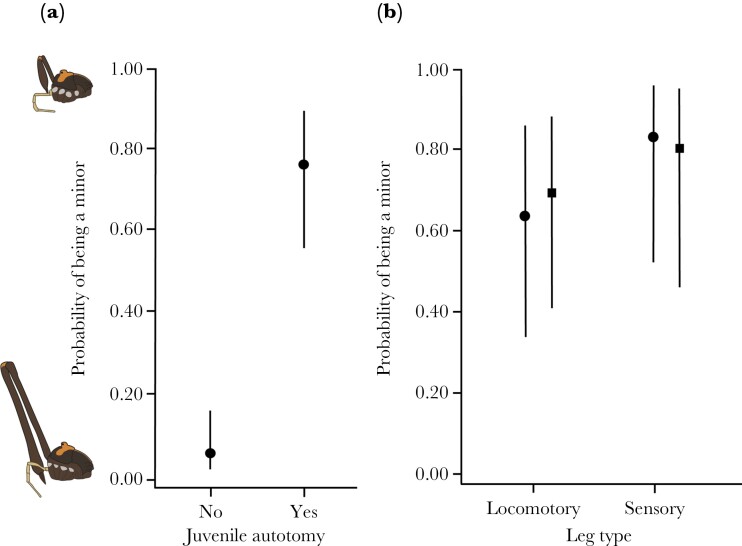
(a) Probability of males of the New Zealand harvestman *Forsteropsalis pureora* becoming a minor (i.e., a gamma morph) as adults depending on the occurrence of juvenile leg autotomy (i.e., a leg loss during development). (b) For males who lost legs during development, the probability of *F*. *pureora* males becoming a minor as adults depending on the type of leg lost. We ran the analyses using two classifications of leg type. In the first (circles), legs III and IV were classified as walking, whereas legs I and II were classified as sensory. In the second (squares), legs I, III, and IV were classified as walking, whereas only legs II were classified as sensory. In both graphics, the higher the probability value, the higher the chance of the male becoming a minor when adult; the lower the probability value, the higher the chance of the male becoming a major (i.e., an alpha or a beta) when adult. Vertical lines represent the 95% confidence interval.

The relative frequency of juvenile autotomies among males of the minor morph was 82.6% (*N* = 19 of 23) and among males of the major morph was 9.5% (*N* = 6 of 63). All major males with juvenile autotomies lost only one leg (*N* = 6), while minor males with juvenile autotomies lost either one (*N* = 17) or two legs (*N* = 2). Juvenile autotomy was a strong predictor of male morph determination: males that lost at least one leg had a 45 times higher probability of becoming a minor, when adults, than males that lost no legs (Odds ratio_(Yes/No)_ = 45.125 ± 31.485, *z*-ratio = 5.460, *P* < 0.001, Figure 5a). Leg type lost during development had no influence on morph determination, regardless of the classification of legs I as a locomotory (odds ratio_(Walk/Sens)_ = 0.562 ± 0.559, z-ratio = −0.579, *P*-value = 0.562) or as a sensory appendage (odds ratio_(Walk/Sens)_ = 0.350 ± 0.349, *z*-ratio = −1.054, *P* = 0.292; Figure 5b). The probability of a juvenile male becoming a minor as an adult was slightly higher when the missing leg was sensorial, but the 95% confidence interval of the estimates overlaps with those of the locomotory legs (Figure 5b). For both leg types, juvenile autotomy was associated with a high probability (locomotory > 0.6, sensory > 0.8) of a male becoming a minor when adult (Figure 5b).

## DISCUSSION

Here, we show that juvenile leg autotomy is related to adult morph in *Forsteropsalis pureora*, a harvestman with marked weapon polymorphism. This finding suggests that male alternative reproductive tactics (ARTs) in this species are a conditional strategy. Conditional strategies have been shown for several species to explain the reproductive tactics of males unlikely to be competitive in traditional male contests (e.g., bluegill sunfish: Gross 1991; crickets: Rowell and Cade 1993; harvestmen: Buzatto et al. 2011; dung beetles: Emlen et al. 2012). In the case of *F*. *pureora*, leg loss during development may, directly or indirectly, play a part in a conditional strategy, influencing the morph a male becomes as an adult. There are many ways that the environment juveniles experience can lead to carryover effects which may influence morphology throughout development, final adult phenotype, performance, and fitness (reviewed in O’Connor et al. 2014 and Moore and Martin 2019). In dragonflies, for instance, some males invest more time in foraging activity during development, leading to higher body condition as juveniles (Stoks 1998) and larger wing ornaments at adulthood (Moore and Martin 2018). However, increased foraging activity also increases predation risk during development (Moore and Martin 2018). Similar trade-offs between predator exposure and foraging may occur in *F*. *pureora* males if they increase their exposure to predators during development while acquiring the nutritional resources necessary to invest in exaggerated weapons at adulthood. The loss of legs owing to increased risk of predation may ultimately make developing into a major (alpha or beta) either unattainable due to lower access to nutritional resources or a poor strategy because males are at an immediate disadvantage in contests without fully intact legs.

Our results also show that there is no difference between the type of missing leg (locomotory or sensorial) on morph determination. Legs II are primarily used for sensory functions (Willemart et al. 2009), such as food detection, so the loss of these legs during development could reduce foraging success. In turn, the loss of a locomotory leg could reduce the locomotor performance, which may have negative effects on the foraging success of a species that relies heavily on active hunting, such as *F*. *pureora* (Powell et al. 2021a). Unfortunately, all studies on the effect the type of missing leg has on the foraging success and locomotor performance of autotomized harvestmen have been done with adults (e.g., Escalante et al. 2013; Domínguez et al. 2016), and we do not know if the results obtained for adults also apply to juveniles, which are markedly different in terms of size, habitat use, diet, and foraging behavior (Acosta and Machado 2007). Based on our results, however, we suggest that the loss of any type of leg is detrimental to the foraging success and/or locomotor performance of juvenile males of *F*. *pureora*. Therefore, a poor diet due to the lower foraging success potentially caused by leg autotomy during development may decrease the chances that a male will reach his own conditional threshold (sensu Tomkins and Hazel 2007), and consequently would indirectly affect the likelihood of becoming a major morph at adulthood.

While leg loss is likely to reduce the foraging success of males, it is also possible that autotomy during development causes a direct ontogenetic switch signaled by leg loss itself. A known ontogenetic switch in arthropods is *acceleration*, which occurs when males molt to adulthood early, even skipping developmental instars (Taborsky 1998; Kelly and Adams 2010). Males may mature earlier to “make the best of a bad job” (Dawkins 1980) as a scrambling minor morph when there is no longer the potential to be a competitive fighting major morph. For example, the tree wētā *Hemideina crassidens* has three male morphs driven by ontogenetic acceleration and hypermorphosis (Kelly and Adams 2010) and, in the amphipod *Jassa marmorata*, majors take longer to mature than minors (Kurdziel and Knowles 2002). Developing into a major morph may be a poor strategy if fully intact legs are important for winning contests, which is likely to be the case in this species. In the harvestman *Leiobunum vittatum* (without exaggerated weaponry), males engage in contests for territory possession, and individuals with all eight legs are more likely to win these contests than males that had autotomized legs (Macías-Ordóñez 1997). In *F*. *pureora*, it is likely that males would pay even higher costs of missing limbs while wielding large cumbersome weaponry in contests compared with other long-legged species that do not have exaggerated weaponry, such as representatives of the families Phalangiidae and Sclerosomatidae.

Limb autotomy may either accelerate or delay molting in many arthropod taxa (reviewed in Fleming et al. 2007). It is known that ecdysteroid (molting hormone) titers rise after leg damage in species belonging to distantly related clades, such as cockroaches (Roberts et al. 1983) and crabs (Mykles 2001), suggesting a widespread physiological phenomenon in arthropods. Male weaponry in beetles is well-known to be an endocrine-mediated trait (Emlen and Nijhout 1999; Moczek and Emlen 1999; Gotoh et al. 2011; Emlen et al. 2012), and previous studies suggest that weaponry in harvestmen is also driven by compartmentalized hormone regulation (reviewed in Buzatto and Machado 2014). Though harvestmen are unable to regenerate a limb, if leg autotomy during the juvenile stage initiates an increase in ecdysteroid hormones, this could directly act on the same system that regulates weapon and body size and consequently determine male morph at adulthood.

An alternative hypothesis to explain the pattern reported here is that males may have underlying genetic polymorphisms correlated with variable ARTs and consistent behaviors that persist throughout the lifetime (Sih et al. 2004). For example, rovers (that move more) and sitters (that move less) in *Drosophila* fruit flies have consistently different activity levels at the larval stage that follow into the adult stage (Tortorici and Bell 1988; de Belle et al. 1989; Pereira and Sokolowski 1993). In *F*. *pureora*, increased activity in males that will become scrambling minors as adults may begin upon hatching and expose these males to more predators, leading to increased leg loss via leg autotomy. However, in another study with this same harvestman species, we found no evidence that the scrambling mating tactic of the minors, which involves more movement than the resource-guarding mating tactic of the majors, leads to a higher frequency of leg autotomy during adulthood (Powell et al. 2021b).

Another possibility is that mortality could be higher in alpha and beta males that experience leg loss as juveniles. If autotomized males that will later become majors die during development, they would not be captured in the sample of adult males. This differential mortality of the morphs in the juvenile stage could be responsible for the pattern reported here. However, Powell et al. (2021b) found no differences in the willingness of adult males of different morphs to autotomize legs when autotomy was experimentally induced with a predator simulation. If the same happens with juvenile males, we could discard differential mortality during development as a reasonable explanation for our findings.

A final explanation for our results is that if harvestmen are already in poor condition as juveniles due to low-quality nutrition or other early-life influences, more molting accidents may occur as they do in taxa such as stick insects (Maginnis 2006b) and leaf-footed bugs (Emberts et al. 2020). Legs lost during a failed molt could explain the pattern found here, i.e., being in poor condition could cause leg autotomy rather than be a result of leg autotomy. We stress, however, that the ecology and behavior of juvenile harvestmen remains poorly studied because they have secretive habits and, as with other arthropods, cannot be followed in a long-term mark-recapture study because they lose their mark after molting. The best way of testing the hypotheses raised in our study is to track juveniles over time under suitable laboratory conditions—a task that has already been tried with many harvestman species but has rarely been successful (E.C. Powell, C. Painting, G. Machado, and G. Holwell, unpublished data).

In conclusion, we have shown correlative evidence that early leg autotomy is related to adult weapon polymorphism. Losing legs during the adult stage is known to impose costs for locomotion, foraging, and competitive ability in many arthropod taxa (reviewed in Fleming et al. 2007 and Emberts et al. 2019). In the harvestman *F*. *pureora*, leg loss during the juvenile stage seems to represent an even more severe cost: by altering the developmental trajectory of the males, it may also affect their lifetime reproductive success if minors have lower mating or fertilization success than majors during the breeding season. Thus, our study opens three fruitful lines for future investigation: (1) What is the proximate mechanism that relates leg loss during the juvenile stage and male morph at adulthood? (2) What are the consequences of leg loss for the reproductive success of the males? (3) How often do external ecological effects drive morph frequency in other taxa? Answers to these questions may shed light on the evolution and maintenance of an extreme form of male polymorphism in arthropods.

## Data Availability

Analyses reported in this article can be reproduced using the data provided by Powell et al. (2023).

## Notes

We thank L. Gomes, C. Selleck, and N. Willmott for assistance in the field and lab. The Stubbs family and the NZ Department of Conservation (50566-RES) provided land access. We thank N. Willmott, L. Taylor, and L. Lietzenmayer for feedback on earlier versions of the manuscript. S. García Hernández provided helpful discussion regarding the statistical analyses. Funding was provided by a RSNZ Marsden grant to GIH (#15-UOA-241), the Entomological Society of New Zealand (to ECP-2018), the Australasian Society for the Study of Animal Behaviour (to ECP-2018), and the School of Biological Sciences at the University of Auckland. GM is supported by a research grant of the Conselho Nacional de Desenvolvimento Científico e Tecnológico (306550/2014-8).
